# Recent Advances in Nanoparticle-Mediated Co-Delivery System: A Promising Strategy in Medical and Agricultural Field

**DOI:** 10.3390/ijms24065121

**Published:** 2023-03-07

**Authors:** Mingshan Li, Xiaowei Sun, Meizhen Yin, Jie Shen, Shuo Yan

**Affiliations:** 1 Department of Plant Biosecurity and MARA Key Laboratory of Surveillance and Management for Plant Quarantine Pests, College of Plant Protection, China Agricultural University, Beijing 100193, China; 2 State Key Laboratory of Chemical Resource Engineering, Beijing Laboratory of Biomedical Materials, Beijing University of Chemical Technology, Beijing 100029, China

**Keywords:** cancer therapy, co-delivery system, nanoparticle, nanopesticide, RNA pesticide

## Abstract

Drug and gene delivery systems mediated by nanoparticles have been widely studied for life science in the past decade. The application of nano-delivery systems can dramatically improve the stability and delivery efficiency of carried ingredients, overcoming the defects of administration routes in cancer therapy, and possibly maintaining the sustainability of agricultural systems. However, delivery of a drug or gene alone sometimes cannot achieve a satisfactory effect. The nanoparticle-mediated co-delivery system can load multiple drugs and genes simultaneously, and improve the effectiveness of each component, thus amplifying efficacy and exhibiting synergistic effects in cancer therapy and pest management. The co-delivery system has been widely reported in the medical field, and studies on its application in the agricultural field have recently begun to emerge. In this progress report, we summarize recent progress in the preparation and application of drug and gene co-delivery systems and discuss the remaining challenges and future perspectives in the design and fabrication.

## 1. Introduction

Over the past decade, nanotechnology has been at the forefront of rapid advances in fields as diverse as medicine, electronics, aerospace, life science, and agriculture [1,2]. The application of nanomaterials can break through the bottleneck of many traditional crafts and provide strong technical supports for nano-delivery platform, thus becoming a research hotspot in the fields of medicine and modern agriculture [3,4,5]. Since the first research on the delivery of drugs by nanomaterials, there have been numerous reports of the application of nanomaterials to deliver active ingredients (AIs) [6,7,8]. To date, many nanomaterials are employed for a nano-delivery system due to their unique physicochemical properties, such as controllable size, low cytotoxicity, enhanced activity of carried ingredients, and breaking the biofilm barrier. For example, polymeric NPs are fabricated from natural and synthetic polymers and are characterized by low cost and biodegradability [9]. Lipids are amphiphilic molecules consisting of a polar head group, a hydrophobic tail, and an intermediate linker [10]. Inorganic NPs are usually synthesized by chemical methods using heavy metal or inorganic material, such as mesoporous silica NPs [11,12], iron oxide NPs [13], gold NPs [14], and quantum dots, etc. [15]. Recently, plants or crops have also been used as feed stocks to develop green synthetic methods [16,17]. Multiple nanoparticles (NPs) have been designed and evaluated as carriers to deliver small molecule drugs for medical or agriculture field, including polymeric NPs, lipid NPs and other inorganic NPs [18,19]. In addition, NPs can deliver various nucleic acid molecules, proteins or photosensitizers, which have been extensively investigated [20,21,22].

Chemotherapy, biological therapy, and radiation therapy are the main forms of cancer treatment, and the former is also considered to be one of the most effective methods in clinical practice [23]. In chemotherapy, patients are often treated with cytotoxic drugs to kill cancer cells [24]. Biological therapy involves the application of biomacromolecules such as nucleic acids to inhibit specific molecules that affect tumor growth [25]. However, the use of chemotherapeutic agents is limited by three major limitations, such as poor water solubility, poor bioavailability, and toxicity of normal tissues [26]. Poor solubility and bioavailability often result in irregular biodistribution and systemic toxicity of chemotherapeutic drugs, which in turn affect normal cells. Multidrug resistance (MDR) caused by long-term and continuous administration is considered as a harmful consequence [27,28,29]. Thus, after extensive attempts, researchers have developed multifunctional vectors that can precisely deliver therapeutic drugs to the site of action. Currently, new therapeutic strategies have been developed to improve treatment efficiency and reduce costs and side effects [30,31,32].

With nano-delivery platforms, small molecule drugs or nucleic acid molecules can be efficiently transported to target tissues without degradation [7,33]. However, single delivery of chemotherapy targeting one pathway is usually not enough, and multiple reasons (such as MDR) hinder the development of effective and long-lasting cancer treatments. Therefore, the combination of different treatments (delivery of genes or drugs) has been proposed as a more ideal cancer treatment strategy and widely studied [34,35,36]. Co-delivery systems can improve the pharmacokinetics and physicochemical properties of therapeutic drugs and improve the efficacy of combination therapy through targeted design of drug delivery regimens [37]. Many combination applications have been designed to achieve synergistic therapeutic effect, and the co-delivery of multiple AIs in the same nanocarrier may achieve desirable effects [38].

Pesticides play a vital role in defending against biological disasters and promoting crop productivity [39]. Traditional pesticides are synthetic organic compounds with high hydrophobicity, which is inconvenient to apply. Meanwhile, traditional processing and formulation requires organic solvents which further poses environmental pollution and biosafety risks [40,41]. Therefore, there is an urgency in scientific use of pesticides and improve the control efficacy of plant diseases and insect pests for green food production. Nanomaterials can be used as substitutes for organic solvents in processing and formulation. Currently, nano-enabled pesticides (nanopesticides) are considered to be less than 1000 nm in size, including insecticides, fungicides, herbicides, and rodenticides, as well as plant immune inducers, plant growth regulators and other AIs that can improve the resistance of plants [42,43]. For precision agriculture, nanopesticides are prepared in different formats of NPs, which show a variety of appealing characteristics, including long-term stability and duration, controlled and stimulation-regulated release rates, increased AI solubility, and improved adhesion to crops, etc. [44,45,46,47]. Recently, a review provided a comprehensive analysis of nanopesticides in controlling agricultural pests compared with their non-nanoscale analogues from 500 journal articles [48]. The overall efficacy of nanopesticides against target organisms is 31% higher, and the toxicity of nanopesticides toward non-target organisms is 43% lower, highlighting that nanopesticides are potentially more efficient, sustainable, and have a lower adverse impact on the environment.

In this review article, we primarily focus on nanoparticle-mediated co-delivery systems. Combining our own work with evidence from the literatures, we highlight the importance of NPs in delivery and co-delivery systems, summarize the latest research and insights in the field of co-delivery, and hope that we will provide some new ideas and stimulate more efforts to promote the widespread use of nano-delivery system in the medical and agricultural field.

## 2. Co-Delivery System in Medical Field

**Figure 1:**
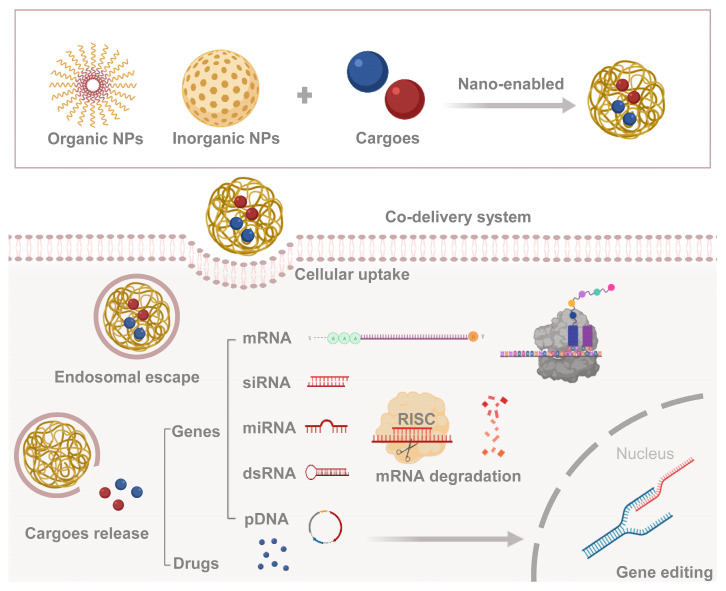
Combination route and mechanism of co-delivery system. Cargoes (genes or drugs) are encapsulated in nanoparticles, and then delivered into the cytoplasm through endosomal escape.

Various NPs have been examined to design novel co-delivery systems, which can be divided into inorganic-based NPs and organic-based NPs. The former mostly includes mesoporous silica NPs, iron oxide NPs, metallic NPs (copper, gold, or silver), quantum dots, etc. The latter includes polymeric micelles, polymeric NPs, liposomes, dendrimers, etc. Recent advances in the development of NPs suggest that these systems can be designed to protect and deliver AIs with different types and sizes, ranging from chemical small molecules to biological macromolecules, and from hydrophilic to hydrophobic agents [38]. The drugs and/or genes (cargoes) are enabled by NPs for efficient cellular uptake and arrive at the target after the endosomal escape to take effect separately (Figure 1). In addition to many types of drugs, nucleic acid molecules come in many varieties, including messenger RNA (mRNA) which is decoded into peptides or proteins; microRNA (miRNA), short interfering RNA (siRNA), and double-stranded RNA (dsRNA) that can induce gene silencing; and plasmid DNA (pDNA) that gets further expression in the nucleus, etc.

### 2.1. Co-Delivery of Drugs

Based on the achievements obtained from the delivery of single chemical drug, co-delivery of two different chemical drugs has been developed and clinically applied to treat different types of cancers [49,50,51]. Compared with monotherapy, combination therapy can not only reduce the possibility of tumor resistance to drugs, but also alleviate the side effects of drugs by reducing the dose of drugs. Different NPs are designed for delivery because of the different physical, chemical and biological properties of these therapeutic agents. Current studies have shown that the delivery of two chemical drugs in the same nanocarrier is much more efficient than a system that delivers a single drug [50,52]. Meanwhile, nanocarriers can improve the water solubility and delivery efficiency of hydrophobic drugs in vivo.

On this basis, co-delivery of other chemotherapeutic drugs or natural active products also achieves synergistic therapeutic effect [53]. Chao and co-workers reported a mesoporous magnetite ferrite NP as an inorganic drug carrier, which can efficiently encapsulate hydrophobic drug (rifampin) and simultaneous co-load hydrophilic drug (isoniazide) [54]. Besides, the prepared NPs exhibit excellent biocompatibility and cellular uptake, which can enhance drug loading capacity and solve the delivery problem of hydrophobic drug molecules [55]. Karimifard et al. fabricated chitosan-adorned niosome nanocarriers for co-delivery of doxorubicin (DOX) and vincristine to reduce drug dose and overcome MDR [56]. The complex effectively inhibits cell migration and induces the apoptosis of breast cancer cells. In addition, the complex shows significant drug release in acidic pH compared to physiological pH, decreasing the adverse off-target effects on normal cells. The overexpression of the drug efflux pumping on the cell membrane is one of the main mechanisms causing MDR, that limits the chemotherapy efficacy [57]. For example, P-glycoprotein (P-gp) encoded by the mdr-1 gene is a key protein on cell membranes and a major drug efflux pump that pumps the drug out of tumor cells. Tian et al. co-delivered heparin and quercetin to breast cancer cells, achieving targeted combination chemotherapy and MDR reversal [58]. The complex can significantly block tumor lymphatic formation and inhibit the expression of P-gp in tumor cells.

In addition to co-delivery of two chemotherapy drugs, co-delivery of drugs and other cargos has also been studied. For example, Hu et al. developed the co-delivery system of the hydrophobic chemotherapeutic drug paclitaxel and biomacromolecule interleukin-12 (IL-12) based on the mPEG-Dlinkm-PDLLA [59]. The NPs are enriched in the tumor site, which can significantly inhibit the growth and metastasis of breast cancer cells 4T1 and prolong the overall survival of tumor-bearing mice.

### 2.2. Co-Delivery of Genes

Nucleic acid-based gene therapy is based on therapeutic molecules DNA or RNA, which aims to achieve multiple goals in vivo, including (1) deliver siRNA, miRNA or dsRNA for gene down regulations; (2) deliver pDNA or mRNA for gene over expression [60,61]. Co-delivery of the nucleic acids has the potential to regulate target gene expression level, hence changing protein content and even disease development. Similar to co-delivery of antitumor drugs, different formulations containing various nucleic acid molecules have been screened for overcoming MDR [62]. Some researchers have explored the co-delivery of dual-gene nanoplatforms, such as siRNA-siRNA, pDNA-siRNA, siRNA-miRNA, etc., to treat various diseases related to genetic disorders or cancers [59,60,61].

In 2013, Tabernero et al. used lipid NPs to co-deliver two modified siRNAs and performed the first human clinical trials [63]. Ball et al. established the co-delivery system of siRNA and mRNA based on the same lipid NP that can enhance the efficacy of both agents in vitro and in vivo [64]. NPs co-delivering siRNA and mRNA can mediate significantly higher levels of gene silencing compared to NPs loading siRNA alone. When the same set of cells is assessed for mRNA delivery, the co-delivery system again produces better results. Yang et al. used nano-carriers to co-deliver *K-ras* and *Notch* siRNA [65]. This strategy increases the sensitivity of pancreatic cancer cells to the chemotherapy drug gemcitabine and also helps to resolve MDR. Wang et al. designed and constructed liposomal NPs loaded with both *p38α MAPK* and *p65* siRNA [66]. The complex efficiently silences two genes, and eventually alleviates the proteinuria and inflammation in mouse IgAN models. This suggests that co-delivery of nucleic acids plays a role not only in cancer treatment, but also in other disease treatment. With the development and optimization of gene therapy, the CRISPR/Cas system has been studied and developed over years, and it has the potential to enable true cure therapies that fight disease at the DNA level and address its origin rather than just treating its symptoms [67]. The non-viral delivery (nanoparticle delivery system) has been studied and widely used in gene editing, and NPs can effectively deliver CRISPR/Cas9 systems into targeted cells [68,69]. For cancer therapy, Cas9 protein/mRNA/pDNA and single guide RNA (sgRNA) co-delivery system have been designed to knock out tumor-related genes and suppress tumor growth. As an example, Wang et al. developed PEGylated NPs co-delivering Cas9 expression plasmids and sgRNAs, and the gene editing efficiency can reach 35%, which results in significant tumor suppression (higher than 71%) and improves the survival rate of tumor-bearing mice (60%) [70].

### 2.3. Co-Delivery of Genes and Drugs

Although many effective research studies and treatments have been made, nucleic acids face the same problems with cancer heterogeneity and adaptive resistance as traditional small molecule drugs in cancer therapy. With the achievements obtained from the fields of chemotherapy and gene therapy, co-delivery of drugs and genes has attracted wide attention in combination therapy due to its synergistic therapeutic effects [71,72,73]. The general incentive behind the co-delivery system is to disrupt MDR signaling pathways. For example, the combination of anticancer drugs and siRNA has great potential in cancer treatment to achieve synergistic effect and overcomes the hurdlers of using a single drug [74,75]. Zhang et al. reported the graft copolymer-based co-delivery of DOX and siRNA targeted P-gp, that exhibited good effects on reversing MDR and synergistic cancer therapy [76]. This co-delivery can down regulate the expression of P-gp, enhance the cytotoxicity of DOX, and suppress the growth of tumor more effectively than free DOX or DOX/NPs complex in tumor-bearing mice. Similarly, Joshi and co-workers fabricated the hypoxia-sensitive micellar NPs for co-delivery of DOX and siRNA targeted P-gp. Under hypoxic conditions, this combination was 80% cytotoxic in monolayer cells and 20% cytotoxic in spherical cells [77]. Meanwhile, researchers used the gold NPs for delivering both DOX and morpholino AONs, which is one of the modified gene silencing DNA analogs [14]. This complex provides enhanced intracellular uptake of DOX, and co-delivery of morpholino and DOX shows better treatment efficiency compared to the free drug.

In addition to the treatment regimen of reversing MDR, induction of tumor apoptosis through co-delivery of chemotherapy drugs and therapeutic genes is another possible cancer treatment strategy. Recently, a co-delivery system was designed to simultaneously deliver curcumin and *p53* DNA to enhance the sensitivity of drug-resistant ovarian cancer cells to cisplatin [78]. In a different example, a polycationic brush was used as a nanocarrier for co-delivery DOX and pDNA of *p53* [79]. The obtained DOX-NPs/pDNA complexes can transport DOX and pDNA into the same cell. The synergistic delivery of DOX and *p53* genes enhances the cell growth inhibition and reduces the dose of DOX.

## 3. Co-Delivery System in Agricultural Field

In agricultural and environmental fields, some nanoparticles can be used alone due to their own properties [80]. Metal oxides TiO_2_ have been shown to have excellent dye degradation activity and can be applied for environmental remediation [16,81]. Biosynthesized AuNPs modulated the accumulation of nitric oxide and induced salt stress tolerance in wheat plants [82]. Meanwhile, NPs can be directly used as nanopesticides due to their antibacterial or insecticidal properties [81]. For example, copper oxide and calcium oxide NPs can be used to control *Spodoptera littoralis* [83]. Bharani et al. synthesized nanosilver with a good control effect against *Spodoptera litura* [84]. NPs exhibit not only biotoxic properties but also plant disease resistance. The use of silver NPs can control tomato early blight and increase the fresh weight of tomato by 32.58% [85]. Furthermore, NPs can load AIs by means of adsorption, entrapment, and encapsulation to prepare nanocapsule, nanosphere, nanomicelle, and other formulations [86].

### 3.1. Nanoparticles Deliver Pesticides (Drugs)

Nanopesticides are similar to other common pesticide formulations in that they help to improve the apparent solubility of the insoluble AIs, or release the AIs in a slow or targeted manner, thereby protecting them from premature degradation [87,88,89]. For nanopesticides composition, AIs can be loaded on the inorganic NPs surface, incorporated into the pores of porous NPs or conjugated with polymer. The high surface-to-volume ratio of silica NPs has been widely used as nanofertilizers and nanopesticides [90,91]. In a foliar nitrogen fertilizer study, sea urchin-like micro- nanostructured hollow silica spheres with 500 nm particle diameter were used to load a nitrogen fertilizer to improve the utilization rate on plant leaves [92]. Compared with traditional fertilizer, the utilization rate of nanofertilizer increased by 2.29 times, and the adhesion ability of nanofertilizer on peanut and corn leaves increased by 5.9 times and 2.2 times, respectively. Zhu et al. synthesized mesoporous silica NPs to deliver fenoxanil into rice plants, suggesting that the distribution behavior of pesticides in plants can be regulated by NPs [93].

Polymeric NPs are of significant interest for encapsulation of pesticides due to many unique features such as renewable, biodegradable, low cost, and environmental responsibility [94]. Yan et al. used a polymeric NP (Star polycation, SPc) to assemble with botanical pesticide matrine, reducing its particle size to 10 nm in aqueous solution and amplifying its bioactivity by about 20% in vitro and in vivo [95]. The SPc can not only increase the bioactivity of loaded pesticides, but also reduce pesticide residue [89,96]. The SPc can also assemble with calcium glycinate to prepare a calcium nutrition nanoagent with nanoscale size (17.72 nm), thus enhancing transport and antiviral immunity [97]. The calcium transport is accelerated into tomato leaves and the protective effect of calcium glycinate is remarkably improved toward tomato mosaic virus. Furthermore, the SPc can activate the endocytosis pathway of plants to amplify the defense responses induced by chitosan elicitor, and NP-loaded chitosan exhibit enhanced control effects against potato late blight [98].

NPs can greatly improve the environmental stability of AIs and build a controlled release system of agents that respond to external pH, enzyme, light, temperature, and other factors [99]. The stimulus-responsive nanocarriers typically employ widely available and biodegradable natural polymers including ethyl cellulose and starch. Liu et al. developed a composite that chemically functionalized chitosan and attapulgite clay as pesticide carriers capable of responding to UV-accelerated release [100]. The release of the pesticide under UV light stimulation is 3.5 times that under natural light, demonstrating a good performance of light-controlled release of the smartly engineered pesticide. Ren et al. used interface polymerization to combine modified biochar and polyurea microcapsules to co-encapsulate allyl isothiocyanate, developing a model fumigant for controlled release [101]. It shows potent bioactivity against soil-borne pathogens and weeds, and further minimizes fumigant usage. The controlled release systems reduce the dosage and frequency of pesticide application, thus improving the utilization rate of pesticides.

### 3.2. Nanoparticles Deliver Nucleic Pesticides (Genes)

RNA interference (RNAi) is a conserved regulatory mechanism mediated by the siRNA pathway, microRNA pathway, and Piwi-interacting RNA pathway, which can silence or inhibit the expression of target genes [102,103,104]. For nanopesticides, the addition of NPs enhances the stability of nucleic acid molecules and makes them free from degradation. The lipid formulation of dsRNA is protected from the degradation by endonucleases present in Sf9 cell conditioned medium, hemolymph, and mid-intestinal cavity contents of *Spodoptera frugiperda* [105]. For another example, SPc and perylenediimide-cored cationic dendrimer can prevent dsRNA from degradation by RNase A and hemolymph of aphids and fall armyworms [106].

In addition to shielding and protecting dsRNA from nuclease degradation in the environment, NPs can also facilitate the transport of dsRNA across the membrane and avoid its degradation in endosomes or lysosomes. For instance, a cationic core–shell fluorescent nanoparticle is able to accelerate endocytosis and deliver DNA across cell membrane for efficient cellular uptake [107]. Lu and co-workers designed the block copolymer poly to form well-defined, core–shell NPs to facilitate its passage through various physiological obstacles and thus prolong the survival time of dsRNA in the digestive tract, so as to enter the midgut cells of *Locusta migratoria* [108]. The SPc can also efficiently deliver dsRNA across the cell membrane and achieve efficient gene silencing [109]. Compared to naked dsRNA, crucial genes regulating endocytosis and exocytosis are remarkably up-regulated in Sf9 cells treated with a dsRNA/SPc complex [106].

RNAi-based strategy has great potential in combatting plant diseases and pests [110,111,112]. Crops can be directly sprayed with dsRNA (spray-induced gene silencing, SIGS) targeting key genes of plant pathogens or pests to induce specific silencing, thus leading to the decline of pest infestation and finally realizing the sustainable eco-friendly pest management [113,114]. A new formulation was developed with the help of a fluorescent NP. The RNA pesticide rapidly penetrates the insect body wall and effectively inhibits gene expression [115]. Meanwhile, Yan et al. applied the dsRNA/SPc formulation targeting *V-ATPaseD* and *chitin synthase1* genes, and direct spray to soybean seedlings infected with *Aphis glycines* results in a good control effect [116]. The combination of NPs and nucleic acid pesticides can improve RNAi efficiency, deformity and mortality, and become a more sustainable pest management strategy (Table 1). With the reduction of dsRNA synthesis cost, Ma et al. further applied the spray method in the field trail, and the control efficacy was as good as expectated [117]. In addition, NPs can also promote the delivery of dsRNA through root tips in *Arabidopsis* [118], which is conducive to irrigation and trunk injection development. At present, there are few studies on improving the RNAi efficiency of phytopathogenic fungi by NPs. Additionally, the types of NPs delivering RNA pesticides for plant disease management are relatively single. Wang et al. used a variety of NPs to deliver RNA fungicide, and the delivery efficiency and protective effect of SPc were the best among them [5]. Table 2 presents selected applications of NPs-mediated RNAi in fungi and viruses.

### 3.3. Application of Co-Delivery System

For synergetic strategy, the co-delivery of multiple pesticides in a single nanocarrier would allow the effective management with low drug concentration, which can help to greatly reduce pesticide residues and environmental contamination. Although pesticide co-delivery systems are rarely reported for agricultural application, efficient and safe co-delivery nanocarriers are necessary for the development of new environmentally friendly pesticides. Suraphan and co-workers utilized premix membrane emulsion combined with S/O/W double emulsion method to further prepare novel AV/CAP co-delivery microcapsule formulations [133]. The administration encapsulated insoluble chlorantraniliprole (CAP) inside the microcapsules and soluble avermectin (AV) into polylactide shells. The particle size of the porous AV/CAP PLA microcapsule is 3.4 µm, and the loading contents of AV and CAP are not obviously different between the co-delivery microcapsules and their corresponding single delivery microcapsules. The co-delivery system shows the lowest LC_50_ value of 18.1 µg mL^−1^ compared to the commercial CAP and AV. This is the first attempt of co-delivery in agriculture, but it does not fall under the category of nanopesticides.

Firstly, our team constructed SPc as a low-cost multifunctional nanocarrier that can co-deliver the dsRNA and pesticide to develop a novel multicomponent nano-pesticide against devastating green peach aphids [134]. The SPc can self-assemble with botanical pesticide matrine, and then complex with dsRNA to form a nano-sized matrine/SPc/dsRNA complex, which can be efficiently delivered into *Drosophila* S2 cells. The dsRNA (ds*hem*) targeting immune gene *hemocytin* leads to efficient gene silencing and a high mortality rate through SPc-based topical application, and the main lethal mechanism is via the down-regulating *hem* gene, resulting in severe bacterial infection. In the field trial, the ds*hem*/SPc complex exhibits short persistence, and the matrine/SPc complex shows slow-acting property, exposing their defects. Interestingly, both initial acting time and persistence of co-delivery complex are remarkably improved, which overcomes the disadvantages of both agents. The synergistic effect of co-delivery system based on NPs has achieved good performance in pest control. The co-administration of thiamethoxam and dsRNA of *synapsin*, both targeting the nervous system, effectively results in the death of melon aphids [135].

## 4. Limitations and Challenges

While significant advances have been made in co-delivery of drugs and/or genes, a number of issues still need to be resolved before applications. First, the safety and regulatory aspects of nanomaterials are widely concerned. Whether they are used as carriers of medical drugs or pesticides, their own cytotoxicity should be very low. In the application of pesticides, a complete risk assessment is necessary for all aspects of pesticidal nanoformulations, including degradation fate, transport, bioaccumulation, adverse effects, and risk to the environment and human health [48]. In addition, NPs carrying multiple AIs simultaneously will increase in particle size, but relevant studies have confirmed that large size molecules are not easy to cross the cell membrane [136]. In order to achieve the desired drug quality and small particle size requirements, more NPs are required to encapsulate the cargoes, which also raises higher requirements for the safety and design methods of co-delivery systems [137].

As for the fabrication of the co-delivery system, designers need to have a clear understanding of drugs and genes of each component, and fully consider the interactions between nanocarrier and cargo, as well as the synergies between loaded reagents [138,139]. For drug and gene co-delivery systems, the biological distribution and pharmacokinetics of the carried agents will be affected due to the huge differences in physicochemical properties of the two components loaded by NPs, such as the difference in molecular weight and hydrophilicity [140]. For example, Tang et al. constructed a pH-sensitive NP to co-deliver DOX and survivin-targeting shRNA for reversing MDR [141]. The co-delivery system increased the DOX accumulation and down-regulated 57.7% survivin expression. However, in vivo biodistribution studies demonstrated that the copolymer remarkably increased tumor accumulation of DOX by more than 10-fold and shRNA by more than 20-fold. The differences in molecular weight, hydrophobicity, and metabolic stability between small molecule drugs and nucleic acids may greatly affect their biological distribution and pharmacokinetics. The co-delivery of drug-nucleic acid combinations is a challenging task because of the obvious differences in the of the two types of agents. Additionally, the loading method of drug molecules and the synergetic strategy of the co-delivery system need to summarize general rules and explore the optimal design route.

## 5. Perspectives in Pesticide

**Figure 2:**
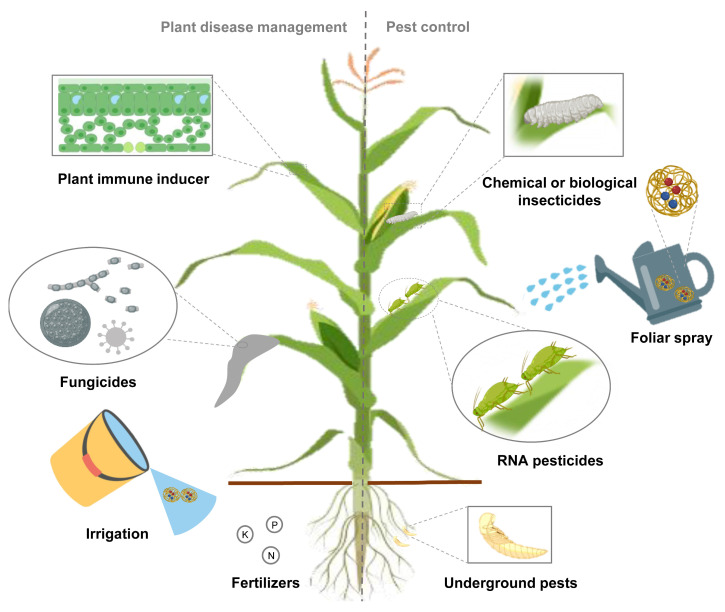
Application of co-delivery system is promising in agricultural field. Fabrication of co-delivery nanopesticide system, assembled with insecticides, fungicides or fertilizers, achieves synergistic effects or multiple aspects of drug administration simultaneously.

The application of NP-based co-delivery systems is mainly divided into synergistic and complementary functions. The co-delivery system, no matter delivering drugs, genes or multiple agents, should be based on solving the bottleneck of pesticide development. Using the synergistic mode of co-delivery system to concentrate on a certain direction, the corresponding drug and nanomaterials can be further reduced and enhanced [135]. For example, the use of co-delivery of conventional pesticides and their corresponding RNA pesticides targeting resistance-related genes avoids the high cost of developing new pesticides and gives traditional pesticides a new lease of life (unpublished data). On the other hand, complementary action in both aspects can reduce the frequency of pesticide application and the dosage of nanomaterials, which is friendlier to the environment [133,134]. For instance, co-delivery of the botanical elicitor cellobiose and dsRNAs of *PiHmp1* + *PiCut3* targeting *Phytophthora infestans* achieves dramatic results. The multicomponent nano-pesticide can not only enter into *P. infestans* more efficiently for gene silencing, but also enhance the systemic resistance of plants. Its protective effect against potato late blight is even higher than that of a widely-used commercial fungicide mancozeb (unpublished data). Although environmental safety of most nanoparticles is still unclear, recent studies have shown that the SPc has some negative effects on non-target organisms at extremely high concentrations [142,143]. To prevent or suppress plant diseases, researchers can develop nanofungicides for plant pathogens; immune inducers and multiple nanofertilizers for plant stress. A variety of insecticides, including chemical or biopesticides and RNA pesticides targeting pests, can be purposefully combined for both above and below ground pests (Figure 2). Multiple application methods including foliar spraying, irrigation, and trunk injection can also be refined to specific applications [113,137]. The production costs of NPs and RNA pesticides should be further reduced, and the application of co-delivery system in the field has been preliminarily realized.

## Figures and Tables

**Table 1:** Applications of NPs-mediated RNA pesticides for insect control.

NPs	Target Pest	Target Genes	Effects	Ref.
block copolymer	*Locust migratoria*	*LmCHS2*	10% died directly and 15% were unable to walk after molting	[108]
cationic dendrimers	*Aphis glycines*	*hemocytin*	80.5% suppression of population density	[115]
polyethylene glycol and chitosan	*Nilaparvata lugens*	*chitin synthetase A*	65.8% mortality	[119]
star polycation	*Myzus persicae*	*vestigial*, *ultrabithorax*	63.3% and 32.2% wing aberration rates	[109]
	*Aphis glycines*	*TREH*, *ATPD*, *ATPE and CHS1*	high mortality up to 81.67%	[116]
	*Myzus persicae*	*ATP-d*, *ATP-G*	61% control efficacy on 3 d	[117]
	*Sogatella furcifera*	*SfEGFR*, *Sfzfh-2*, *SfAbd-A*, *SfAbd-B*	around 70% mortality	[120]
chitosan	*Helicoverpa armigera*	*JHAMT*, *ACHE*	100% mortality	[121]
	*Spodoptera frugiperda*	*IAP*	47% mortality	[122]
liposome	*Euschistus heros*	*V-ATPaseA*, *Muscle* *actin*	45% and 42% mortality	[123]
	*Blattella germanica*	*α-tubulin*	60% mortality	[124]
quantum dot	*Chilo suppressalis*	*G3PDH*	70% mortality	[125]
cerium oxide	*Euschistus heros*	*troponin*	about 80% mortality	[20]

**Table 2:** Applications of NPs-mediated RNA pesticides for plant disease management.

NPs	Host	Pathogen	Target Genes	Effects	Ref.
minicell	strawberry	*Botryotinia fuckeliana*	*Chs3a*, *Chs3b*, *DCL1*, *DCL2*	halted disease progression for 12 days	[126]
star polycation	rice	*Rhizoctonia solani*	*RsAGO1*, *RsAGO2*	the protection time up to 20 days	[5]
nanovesicles	tomato, grape	*Botrytis cinerea*	*Dicer-like 1*, *Dicer-like 2*	extended the protection duration to 10–21 days	[127]
layered double hydroxide (LDH) chitosan	cowpea	common mosaic virus	*CMV2b*	virus protection for at least 20 days	[128]
	cowpea	bean common mosaic virus	*coat protein*	prevent infection for spraying 5d in advance	[129]
	grape, cherry	*Botrytis cinerea*	*erg13*, *erg11*, *erg1*	reduced the decay development by 65 % after 3 weeks	[130]
	maize	*Rhizoctonia solani*	*RsCRZ1*	reducing lesion areas from 30% to 47%	[131]
	tomato	*Botrytis cinerea*	*BcDCL1/2*, *BcVDS*	increased the protection window 3 weeks on tomato leaves	[132]
